# Carbon Ion Radiotherapy: A Review of Clinical Experiences and Preclinical Research, with an Emphasis on DNA Damage/Repair

**DOI:** 10.3390/cancers9060066

**Published:** 2017-06-09

**Authors:** Osama Mohamad, Brock J. Sishc, Janapriya Saha, Arnold Pompos, Asal Rahimi, Michael D. Story, Anthony J. Davis, D.W. Nathan Kim

**Affiliations:** Department of Radiation Oncology, University of Texas Southwestern Medical Center, Dallas, TX 75390, USA; Osama.mohamad@utsouthwestern.edu (O.M.); Brock.Sishc@utsouthwestern.edu (B.J.S.); Janapriya.Saha@utsouthwestern.edu (J.S.); Arnold.Pompos@utsouthwestern.edu (A.P.); Asal.Rahimi@utsouthwestern.edu (A.R.); Michael.Story@utsouthwestern.edu (M.D.S.); Anthony.Davis@utsouthwestern.edu (A.J.D.)

**Keywords:** hadron therapy, radiation oncology, DNA repair, proton therapy, complex DNA damage, carbon therapy

## Abstract

Compared to conventional photon-based external beam radiation (PhXRT), carbon ion radiotherapy (CIRT) has superior dose distribution, higher linear energy transfer (LET), and a higher relative biological effectiveness (RBE). This enhanced RBE is driven by a unique DNA damage signature characterized by clustered lesions that overwhelm the DNA repair capacity of malignant cells. These physical and radiobiological characteristics imbue heavy ions with potent tumoricidal capacity, while having the potential for simultaneously maximally sparing normal tissues. Thus, CIRT could potentially be used to treat some of the most difficult to treat tumors, including those that are hypoxic, radio-resistant, or deep-seated. Clinical data, mostly from Japan and Germany, are promising, with favorable oncologic outcomes and acceptable toxicity. In this manuscript, we review the physical and biological rationales for CIRT, with an emphasis on DNA damage and repair, as well as providing a comprehensive overview of the translational and clinical data using CIRT.

## 1. Introduction

More than two-thirds of cancer patients receive radiation therapy (RT) alone or in combination with other cancer treatment modalities such as surgery or chemotherapy. Most clinical RT utilizes bremsstrahlung photon beams, a form of ionizing radiation (IR), that result from rapid deceleration of typically 4–18 MeV electron beams on high atomic number (Z) targets such as tungsten. While recent advances have led to the development of highly sophisticated photon-based external beam radiation (PhXRT) techniques (including intensity-modulated RT (IMRT), image guided RT (IGRT), and stereotactic body RT (SBRT)) which have resulted in a significant widening of indications and improvement of outcomes, there are still many tumor sites and histologies that remain challenging to cure with PhXRT. It has long been recognized that proton or heavier ion therapy, which uses accelerated charged particles, provide significant physical, biological, and potential clinical benefits over PhXRT [1,2].

The use of heavy charged particles and fast protons for cancer therapy (also collectively termed hadron therapy) was first proposed by Robert Wilson in 1946 [3], and the first treatments using protons and helium ions began in 1954 and 1957 at Lawrence Berkley National Laboratory, respectively [4,5]. Recently, particle therapy facilities have emerged across the world, with over 30 proton centers in operation or under construction in the United States alone, and 10 centers treating patients with carbon ions (C-ions) worldwide [6,7]. As the use of heavy charged particle therapy becomes more widespread, the need for a greater understanding of the biological mechanisms and factors that may affect treatment outcomes becomes more apparent. In this review, we will summarize these physical and biological rationales, as well as, give an overview of the available pre-clinical and clinical data that demonstrate the increased efficacy of heavy ion therapy over conventional PhXRT.

## 2. Rationale for Charged Particle Therapy

### 2.1. Physics Rationale: The Spread-Out Bragg Peak, Enhanced Dose Distribution, Lateral Focusing, Dose Verification, and Superior Linear Energy Transfer

The factors unique to charged particles that can contribute to an overall superior delivery of radiation dose when compared to PhXRT include: (1) spread-out Bragg peak, leading to enhanced dose distribution and lateral focusing; (2) potential for dose verification via available imaging; (3) superior linear energy transfer and; (4) magnetic steering of the ion beams (scanning beams). These properties theoretically should lead to optimal delivery of maximally safe and potentially curative doses of radiation to the tumor while simultaneously sparing at risk adjacent structures.

#### 2.1.1. The Spread-Out Bragg Peak, Enhanced Dose Distribution, and Lateral Focusing

**Figure 1:**
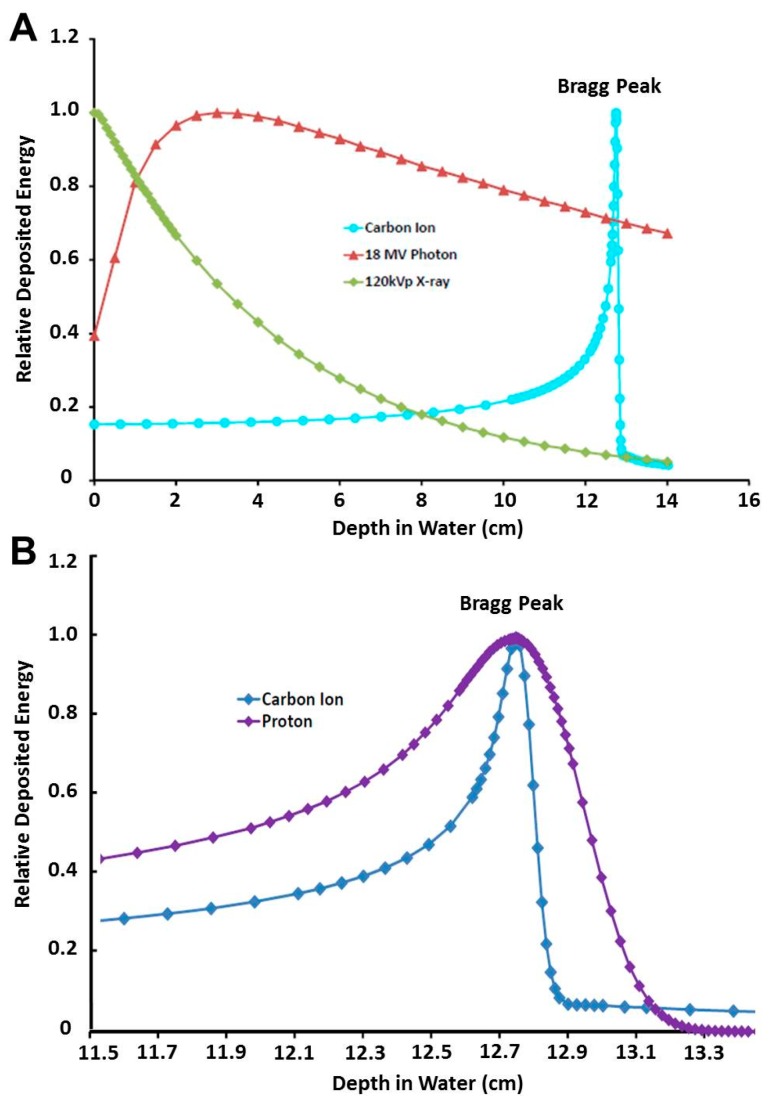
(**A**) Percentage depth dose (PDD) curves comparing carbon ion beams to high (18 MV) and low (120 kVp) energy photon beams. (**B**) Percentage depth dose curves comparing carbon ion to proton beams.

The majority of challenges associated with PhXRT result from the physical characteristics of photon interactions with tissue. The depth dose distribution in tissue rises initially as the secondary electrons liberated by incident photons build up their fluence, as well as the locally deposited dose, to a maximum. Both fluence and dose then decline exponentially as a function of depth and photons are absorbed even beyond the target until they exit the body [8]. This leads to a relatively shallow (maximum depth of about 3–4 cm) depth dose maximum (Figure 1A), which poses a challenge to treating deep-seated tumors without compromising proximal and distal healthy tissues. Additionally, since photons lack electric charge, they can be neither magnetically nor electrically focused and require the use of thick collimators to laterally shape the photon beam to conform to a target. Furthermore, after photons leave the collimator and before they reach the target region, scattering interactions occur, causing lateral spread outside of the collimated field. This results in a lateral penumbra region where radiation dose is deposited in areas where it is not intended. Collectively, these factors contribute to the irradiation of non-tumor normal tissue, increasing the risk of radiation-induced adverse effects, which can potentially limit the amount of curative dose that can be delivered to the tumor. Thus, tumors situated near at-risk structures often cannot be treated to curative doses without a substantial risk of normal tissue toxicities.

In contrast to photons, when charged particles, such as carbon ions, penetrate matter, they immediately start transferring kinetic energy to the medium they traverse [8]. The rate of this energy loss determines how much energy is transferred from the ion to the medium. This energy transfer is quantified as the linear energy transfer (LET) and it increases as the particle slows down until its entire kinetic energy is depleted and the particle comes to rest. This process produces a characteristic depth dose curve where relatively little dose is imparted in the more shallow regions of the track, but increases abruptly and peaks as the particle comes to a stop. The imparted dose falls off dramatically thereafter. This abrupt and drastic deposition of energy in a well-defined range is referred to as the “Bragg peak” [9]. This superior depth dose characteristic of C-ions over both photons and protons is illustrated in Figure 1A,B, respectively.

The depth of penetration is a function of the initial kinetic energy and particle charge. More energetic particles are able to penetrate deeper, and larger charged particles penetrate less deep with the same initial kinetic energy. If one utilizes beams of particles with different initial kinetic energies that are appropriately weighted with respect to each other, a region of uniform dose (or in fact any shaped physical dose distribution) in the depth (or beam) direction can be created to cover the treated lesion. Charged particles have an additional significant advantage with respect to conventional X-rays; they can be magnetically steered rather than physically collimated. If a narrow beam of charged particles is deflected in the lateral direction, the whole tumor can be painted with the planned radiation dose. This, combined with the above mentioned longitudinal spread, results in what is termed as the “spread-out Bragg peak” (SOBP). It grants clinicians the ability to “paint” a tri-dimensional tumor with radiation doses while minimizing the amount of radiation delivered to nearby at-risk structures.

This dose deposition pattern is the foundation of charged particle cancer therapy and the physical properties of the SOBP provide several significant benefits for particle therapy when compared to photons [4]. The first is the enhanced ratio of dose deposited to tumor relative to that deposited in healthy tissue proximal to the tumor. Second, less of the dose is delivered to normal tissues on the back end of the Bragg peak, which allows for additional sparing of normal tissues at the distal edge of the tumor [10,11]. However, due to the stochastic nature of energy loss as ions travel through tissue, not all ions stop exactly at the same depth. This range uncertainty causes a widening of the Bragg peak in the longitudinal direction and is reduced as the atomic mass of the therapeutic ion is increased. A third important contrast of heavy charged particles with photons and protons is the suppressed multiple coulomb scattering they exhibit as they travel through matter. This leads to the sharper lateral penumbra of heavy ion beams, exploited in the clinic by the fact that heavy ion beams can potentially be placed closer to at risk organs laterally, while maintaining a high degree of organ sparing.

#### 2.1.2. Dose Verification

Another potential clinical advantage of hadron therapy over X-rays is that while the energy deposition to tissue via PhXRT is difficult to be directly verified during irradiation using modern imaging techniques, this limitation may be overcome for heavy ions. As heavy ions (e.g., nuclei of carbon atoms) travel through matter, both the ion projectiles and the traversed matter undergo nuclear interactions. Some of these nuclear interactions produce positron emitting nuclei that can be imaged by positron emission tomography (PET) scanners. The downside of these nuclear interactions is the production of low Z fragments that have a large forward momentum, and can travel beyond the range where the incident therapeutic nuclei stop. This overshoot is what causes the existence of a low dose and low LET tail beyond the Bragg peak.

#### 2.1.3. Superior LET

The final potential clinical advantage of hadron therapy is that they utilize heavy charged particles with superior LET values greater than photons or protons. The LET of a particle is determined primarily by its charge and speed. Ionizing radiation can be either high- (densely ionizing) or low-LET (sparsely ionizing). Photons are generally considered low-LET radiation with a broad distribution of dose within tissue peaking relatively near the surface [12]. Heavier charged particles can be either high- or low-LET depending on their speed. At the entrance region in tissue they have lower LET values due to their high speeds, with increasing LET values at lower kinetic energies as the particle comes to rest in deeper regions where the tumor is located. The clinical manifestation of this is that even if proximal tissues receive some dose, at shallower depths, the particles traversing these tissues are of a lower-LET and are therefore less damaging than the high LET portion of the track that is strategically placed into the tumor region. The distribution of dose on nanometer and micrometer scales, resulting from particles with high LET values, is the consequence of large clusters of ionization events along the particle track resulting from direct or collisional events (densely ionizing). This event, which portends a drastically increased micro-dosimetry, results in what is considered to be a much more complex damage to the DNA and to the other relevant biomolecules and, thus, can lead to more consequential biological effects when compared to low-LET radiation.

### 2.2. Biological Rationale: Relative Biological Effectiveness, Complex DNA Damage, and Oxygen Enhancement Ratio

While absorbed dose is a physical unit quantifying the amount of energy deposited as ionization events in tissues, the biological consequences of equivalent absorbed doses resulting from low- vs. high-LET IR can be vastly different. To compare the efficacy of different radiation types, the concept of relative biological effectiveness (RBE) has been developed. RBE is defined as the ratio of the amount of dose from a test radiation required to generate the same biological endpoint (usually cell killing) relative to a reference radiation (usually 250 kVp X-rays or Co-60 γ-rays) [1,13,14]. Therefore, radiation beams with higher RBE values are more effective at producing biological effects at equivalent doses. Photons, by definition are generally regarded as having an RBE of 1, regardless of energy. The commonly reported RBE for protons, calculated at 10% survival, is generally considered ~1.1 [8,15]. However, because past experiments were conducted under a broad range of inconsistent conditions and because of the large variability of results, this value is not considered definitive [8,16,17]. The accepted RBE for C-ions used in clinical RT is generally estimated to be 2.5 to 3, however, values as high as 5 have been reported [18]. RBE is a complex function of LET, particle type, dose per fraction, tissue and cell type, oxygenation state, cell cycle phase, and the endpoint examined. Thus, for C-ions, RBE is variable and increases as a function of depth, with its highest value at the distal edge of the Bragg peak. The distribution of RBE for other ions such as helium, oxygen, and neon corresponds to different portions of the Bragg peak. However, further experimentation and larger data sets are required to obtain the accurate RBE values for protons and heavier ions so that they can be clinically applied into treatment planning systems.

Generally speaking, greater RBE values correlate with increasing LET with a maximum peak of RBE observed at LET values of ~100 keV/μm (for C-ions). The biological mechanism underlying this harkens back to the density of ionization events along the particle track where numerous chemical changes to DNA can occur relatively close to other lesions (both spatially and temporally), resulting is what is termed “complex DNA damage”. IR, whether it is sparsely or densely ionizing, is capable of generating all known types of DNA lesions including single-strand breaks (SSBs), chemically altered base lesions, abasic sites, interstrand crosslinks, intrastrand crosslinks, and most consequentially, double-strand breaks (DSBs) [19,20,21]. Complex DNA damage, which is more commonly found along the densely ionizing track of a high-LET particle, is generally regarded to be refractory to repair as it involves the clustering of multiple types of DNA lesions in close proximity to one another, making it difficult for a single DNA repair pathway to resolve the lesion [22]. This complex damage is likely responsible for a substantial portion of the differences in RBE observed between high and low LET radiation. We will discuss in greater depth the intricacies of the repair of these lesions and potential avenues of therapeutic exploitation in later sections. Since complex DNA damage is difficult to repair, it is believed that heavy ion therapy will effectively kill radio- and chemo-resistant tumors. Increased tumor kill is likely due to the inability of DNA repair pathways to faithfully resolve these complex lesions, leaving them unrepaired or misrepaired, often signaling for apoptosis. Additionally, particle therapy is effective at cell killing irrespective of phase of the cell cycle [23,24], unlike photons, where cells are more resistant in late S and G2 phases [25,26].

In addition to the damage caused to DNA and other relevant biomolecules by direct ionizing events, a significant portion of the damage resulting from IR exposure for both high and low-LET radiations is a result of the radiolysis of water and the generation of reactive oxygen species (ROS). These ROS are able to diffuse throughout an aqueous solution and chemically damage DNA even after the initial exposure and this is commonly referred to as “indirect damage”. One requirement for maximum ROS-related damage is the presence of molecular oxygen, which fixes or makes permanent the damage caused by ROS. Quantifying the effect of oxygen is done through the oxygen enhancement ratio (OER). Similar in concept to RBE, the OER seeks to assess the amount of dose necessary to result in an equivalent biological endpoint with or without the presence of oxygen [18]. The OER for low LET photons and protons is generally estimated at 3, meaning that cell killing is roughly 3-fold greater in normoxic vs. anoxic conditions. In contrast, the estimated OER for carbon and other heavy ions varies with LET and can range from 2.5 to 1.0 depending upon the ion charge and LET [27]. This differential cell killing may be at least partially explained by the increased hypoxia inducible factor-1α (HIF-1α) expression after photon but not C-ion irradiation [28]. Therefore, high LET particles at the appropriate LET (depths) are more effective at killing cells in the hypoxic, necrotic cores of tumors compared to photons, lending particle therapy yet another biological advantage over photons.

## 3. Complex DNA Damage and Repair

### 3.1. Charged Particles and Track Structures

**Figure 2:**
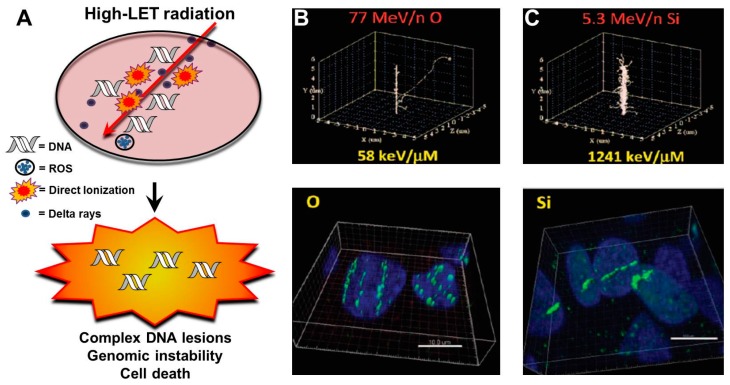
(**A**) High- linear energy transfer (LET) radiation-induced DNA damage consists of clustered lesions which eventually promote genomic instability and, if DNA repair is not successful, cell death. (**B** and **C**) Simulated particle track projections of oxygen (**B**, upper panel) and silicon (**C**, upper panel) beams. Immunofluorescence staining for the DNA damage protein γ-H2AX showing track structures in human fibroblasts after oxygen (**B**, lower panel) and silicon (**C**, lower panel) beams. (Permission was granted to adapt and re-publish these images from Sridharan et al. “Understanding Cancer Development Processes after HZE-Particle Exposure: Roles of ROS, DNA Damage Repair and Inflammation” Radiat Res 2015; 183:1–26 and from Saha et al. “Biological Characterization of the Low-Energy Ions with High-Energy Deposition on Human Cells” Radiat Res 2014; 182:282–291). ROS: reactive oxygen species.

Humans are exposed to high charge and energy particles during hadron therapy with protons and C-ions. Unlike low LET radiation, such as X- or γ-rays, that deposit their energy uniformly over target tissue, heavy charged particles deposit significant amounts of energy along the traversal path constituting a characteristic track structure (Figure 2A–C) [29]. The track structure typically consists of a cylindrical “core” and an outer region denoted as “penumbra”. The bulk of the energy is deposited in the central core of the charged particle track, while the penumbra region consists mainly of sparsely ionizing electrons or δ-rays originating from ionization events in the track core. δ-rays or electrons induce simpler damage that is generally repaired efficiently, unlike charged particle interactions in the core, which are generally more complex, requiring multiple DNA repair pathways to resolve.

### 3.2. LET and Clustered DNA Damage

The degree of DNA lesion complexity directly depends on the LET or ionization density of the radiation [30]. Ionization events caused by photons (low LET) exhibit both a direct and indirect component. Photons can directly deposit their energy uniformly to the DNA molecule, but typically they induce DNA damage by indirectly interacting with biomolecules, resulting in the release of secondary electrons which interact with water molecules to produce free radicals that chemically damage DNA [26]. Typically, low LET irradiation results in simple DNA damage, which is a single DNA lesion within one or two turns of the DNA helix. Conversely, high LET-charged particle radiation primarily induces DNA damage by direct ionizations of the DNA molecule. High LET irradiation leads to a large deposition of energy, resulting in the induction of clustered DNA damage (also termed multiple damage sites (MDS)). This is characterized by multiple DNA lesions within close proximity to one another (one or two turns of the DNA helix) [31,32,33]. DNA lesions found at clustered DNA damage sites can include double-strand breaks (DSBs) and non-DSB lesions like single-strand breaks (SSBs) and oxidative clustered DNA lesions (OCDLs), such as abasic sites, and/or base damages (oxidized purines or pyrimidines) [34,35]. Track structures of charged particle irradiation can be visualized by immunofluorescence techniques utilizing antibodies directed against DNA repair markers including the γ-H2AX and 53BP1 proteins. This allows for the tracking and study of DNA repair events at these break sites. The fact that clustered DNA lesions are refractory to repair can be assessed by the persistence of DNA damage markers γ-H2AX and 53BP1 foci for long periods after IR-induced damage has occurred. Indeed, the close proximity of these lesions was conclusively demonstrated by co-localization of 53BP1, XRCC1, and hOGG1 foci, surrogate markers for DSBs, SSBs, and base damages, respectively, at the sites of clustered DNA damage [22]. Ionizing radiation-induced foci (IRIF) formed after high LET radiation are brighter and larger in dimension than those generated following low LET radiation [36,37]. Although it is true that low LET radiations [38] exhibit a 1:1 ratio of IR-induced DSB to γ-H2AX foci, this is debatable for charged particle radiation. In fact, utilizing super-resolution microscopy, it was recently shown that accelerated C-ions induce densely spaced DSBs and that multiple DSBs constitute an IRIF [39]. The larger γ-H2AX foci (Ø 700–1000 nm) induced following carbon irradiation contained elongated sub-foci (∼100 nm). These sub-foci contained even smaller subfocus elements (Ø 40–60 nm), which were predicted to represent the local chromatin structure of DSB repair units that accumulated at the sites of complex damage. However, whether these sub-foci represent individual DSBs within a large focus is still an open question and warrants further investigation. Along similar lines, utilizing high resolution transmission electron microscopy (TEM) with gold-labeled DNA repair factors, a significantly higher yield of DSBs was reported with high LET carbon ions as compared to low LET photons.

Local decondensation of heterochromatin (HC) along with the bending of foci tracks around heterochromatic regions of chromatin occurs following charged particle damage [40]. Persistent DSBs induced by carbon ion irradiation showed a predominantly HC localization, indicating that local chromatin density at the sites of energy deposition defines the degree of clustering of high LET-induced DNA damage. Since the clustered lesions in euchromatin are efficiently repaired after high-LET carbon ion radiation, the persistence of DNA damage at HC regions is probably due to impinged access of DNA repair machinery to these break sites, requiring extensive mechanisms for chromatin relaxation to repair these breaks. Clustering of these DSBs after high-LET carbon ions makes DNA repair more challenging [41]. More directed studies toward understanding the spatiotemporal repair dynamics of carbon ion induced clustered DNA damage is essential for its continued and expanded utilization as a hadron therapy tool in humans.

### 3.3. Clustered Damage, Chromosomes and DNA Repair

**Figure 3:**
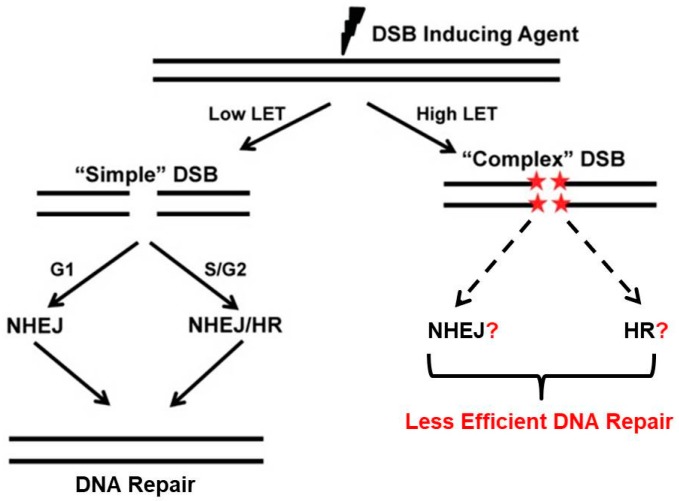
Repair of high- and low-LET radiation-induced DNA damage. Low-LET radiation-induced DNA double-strand breaks (DSBs) are typically repaired by non-homologous end joining (NHEJ) or both NHEJ and homologous recombination (HR) if cells are in S or G2 phases of the cell cycle. The repair of complex DSBs generated by high LET radiation including carbon ions is poorly understood. The less efficient repair response after high-LET radiation leads to DNA damage remaining unrepaired for long periods of time and eventually may promote genome instability and cell death.

In response to clustered DNA damage, cells activate multiple DNA damage response repair pathways. The most toxic of the DNA lesions are DSB, which if left unrepaired or are misrepaired can result in genomic instability or cell death by a number of mechanisms including mitotic catastrophe, apoptosis, or senescence. DSBs are repaired by three pathways: homologous recombination (HR), non-homologous end joining (NHEJ), and alternative end joining (Alt-EJ). Repair of charged particle/high LET radiation-induced complex DNA lesions is not well understood. It is unclear if cells preferentially select a specific pathway to repair DSBs generated by high LET radiation, unlike low LET radiation-induced DSBs, which are typically repaired by NHEJ or both NHEJ and HR if cells are in S or G2 phases of the cell cycle (Figure 3). It has been proposed that complex DSBs generated by high LET irradiation from very high Z particles are repaired by homologous recombination (HR) and not NHEJ in mammalian cells [42]. It is believed that a large number of small DNA fragments generated by charged particle radiation prevents the loading/binding of the Ku heterodimer, an NHEJ factor, onto broken DNA ends, which leads to suppression of NHEJ-mediated repair of DSBs at clustered damage sites [43]. Furthermore, a modulated response of the pro-HR and DNA end resection protein Mre11 to high LET induced DNA ends has also been reported [44]. Early recruitment of the DNA end resection and the DNA strand invasion proteins of the HR pathway along with Rad51 [37] occurs at sites of clustered lesions, indicating that the HR pathway may be favored for the repair of heavy charged particle-induced clustered DNA lesions. This is true as well for proton irradiation [45,46]. Indeed, mammalian cells deficient in HR factors like Rad51 paralogs are sensitive to heavy particle radiation [42]. Nonetheless, recent reports have indicated that NHEJ may play a prominent role in repair of carbon ion-induced damage [47,48]. Reduced survival and cytogenetic differences were also observed between normal and NHEJ-deficient cells after charged particle radiation induced damage [49,50,51]. It has been also shown that clustered DNA damage affects DNA damage response by altering the ataxia telangiectasia mutated (ATM) to ataxia telangiectasia and Rad3 related (ATR) transition at break sites along with persistent ATM/activating transcription factor 2 (ATF2) signaling, a downstream substrate of ATM [52,53].

It is evident that the high RBE of charged particle radiation is due to the abundance of irreparable breaks induced in the cells as compared to low LET radiation [33,54]. However, how these irreparable breaks translate to high cell lethality is not well understood. Heavy ion radiation has been shown to down-regulate pro-survival protein kinase B (AKT) signaling, which leads to activation of pro-death signaling mechanisms such as autophagy and apoptosis [55]. Another potential mechanism for DNA damage response to carbon ion radiotherapy (CIRT) is mitotic catastrophe. In a study of 20 different human cancer cell lines exposed to cisplatin, PhXRT and/or C-ions, apoptosis and senescence were common to all treatments but mitotic catastrophe was differentially triggered by CIRT [56]. The authors argued that the less efficient repair of the more complex DSBs after CIRT leads to aberrant mitosis and subsequent mitotic catastrophe. Similar dependence on mitotic catastrophe was reported in other studies utilizing the radio-resistant HNSCC cell line SQ20B with emphasis on a sub-population of CIRT resistant cells, likely cancer stem-like cells, which are able to escape this death mechanism and renter the cell cycle due to increased self-renewal potential and reduced apoptotic machinery [57,58]. Clustered DNA damage with dense IRIF along the particle track can dramatically increase the probability of chromosomal aberrations compared to X-rays in human lymphocytes [59] and their hematopoietic progenitors [60]. Extreme proximity of DSBs in clustered lesions may also give rise to increased chromosomal rearrangements [61] which was in agreement with computational modeling data [62]. Utilizing cytogenetic techniques like G2 premature chromosome condensation (G2 PCC) and multicolor banding fluorescence in situ hybridization (mFISH), it has been shown that charged particle radiation is more effective at inducing chromosomal aberrations when compared to low LET radiation [63,64,65,66]. Cells harboring unrepaired breaks that move through the cell cycle uninhibited due to lack of proper G2 checkpoint activation propagate chromosomal aberrations or undergo mitotic catastrophe [22]. Many other groups investigated the uniqueness of DNA damage and DNA damage response after heavy particle irradiation. The impact of telomere length [67] and glutathione metabolism/depletion [68], for example, were proposed as potential players in the differential response to particle radiation in contrast to PhXRT.

## 4. Preclinical Research in Carbon Ion Radiotherapy

Given the aforementioned physical, biophysical and biological characteristics of C-ion beams, carbon ion radiotherapy (CIRT) has the potential to improve local control and reduce normal tissue complications in treating cancer patients especially those with deep-seated and traditionally radio-resistant tumors that have poor outcomes with standard therapies. Preclinical studies indicate that CIRT may be able to overcome some of the factors that have traditionally limited the efficacy of standard radiotherapy. For example, tumor heterogeneity is one determinant of local control after radiation. Studies have revealed that CIRT significantly reduces the impact of tumor heterogeneity on treatment efficacy. In one study, rat prostate tumors of varied histologic grades were exposed to C-ions or PhXRT. The difference in local control was less dependent on tumor grade with CIRT compared to PhXRT. Interestingly, RBE increased with increasing tumor grade indicating the suitability of C-ions for treating tumors which have higher failure rates with conventional PhXRT [69]. This increase in RBE is less about the effectiveness of carbon but more about the ineffectiveness of PhXRT with increased tumor grade (heterogeneity). In addition, unlike PhXRT which has been shown to enhance the migratory and invasiveness potential of various cancer cell lines, C-ions have consistently been shown to decrease cell migration, invasion, and matrix metalloproteinase activity across different cell lines and in in vivo tumor models [70,71,72,73,74,75,76,77]. CIRT has also been shown to modulate the immune response to tumors in mice [78,79] but very little work has been done so far on the interaction of heavy particle irradiation with the immune system. Indeed, more preclinical and translational research needs to be done before CIRT achieves its promise. In this section, we will review few preclinical studies aimed at understanding the mechanisms of CIRT in tumors with a special emphasis on DNA damage and repair.

### 4.1. DNA Damage and Repair after CIRT in Select Preclinical Models

DNA damage and cellular repair capacity are major determinants of RBE and cell killing after heavy particle therapy, including C-ions [80]. In a study from the Gunma University Heavy Ion Medical Center, it has been demonstrated, using advanced high-resolution microscopy and 53BP1 staining in cervical cancer tissue, that CIRT induced more complex DSBs with larger and more clustered 53BP1 foci compared to PhXRT. The higher the LET was, the more complex the DNA damage became with larger and more clustered DSBs. This was the first study to show increased complexity of DSBs and its dependence on LET from human samples after CIRT [81]. Similarly, CIRT abrogated the differential radiosensitivity of quiescent vs. proliferating cells in mice growing squamous cell carcinomas. This effect was also dependent on LET [82]. CIRT was also shown to induce irreparable DSBs, and thus cause increased cell kill in central nervous system (CNS) glioma patient-derived stem and non-stem cells [83] and in neuroblastoma and glioblastoma cell lines compared to PhXRT [84]. However, it is important to point out that not all tumors demonstrate this enhanced radio-sensitivity to C-ions. For example, patient-derived glioblastoma cell lines with high chromosomal instability and dysfunctional ATM pathway signaling were shown to be resistant to CIRT [85].

### 4.2. LET-Dependent Differential Expression of DNA Repair Genes

Ionizing radiation activates a complex network of signaling pathways and induces a wide array of transcriptional changes and gene expression. Multiple studies have demonstrated that the genetic response to radiation is dependent on the energy deposited in tissue by the particular radiation species. Many studies have shown that heavy ions induce a greater and differential change in gene expression compared to PhXRT [86,87,88,89]. In all these studies, genes related to cellular metabolism, cell/organelle organization, cell cycle and DNA damage and repair pathways are commonly up-regulated/down-regulated after C-ion exposure. Interestingly, in a study on human bronchial epithelial cells, changes in gene expression profiles were LET-dependent for some genes and LET-independent for others [90]. These studies indicate that CIRT has a more robust impact on gene expression changes, compared to PhXRT. However, additional studies are required to further elucidate the impact of these findings and to demonstrate the LET dependence in clinical studies.

### 4.3. CIRT and Chemotherapy

Conventional PhXRT has incorporated chemotherapy neoadjuvantly, concomitantly, and adjuvantly in different clinical settings with variable outcomes. Indeed, many chemotherapeutic agents such as cisplatin synergistically increase the efficacy of PhXRT. Very little is known, however, about the safety and efficacy of combining chemotherapy with C-ions. Combining CIRT with chemotherapy has only been used in a few patient cohorts undergoing CIRT [91,92,93]. Generally, chemotherapy demonstrated additive cytotoxicity when combined with CIRT in in vitro experiments [94,95,96,97,98]. For example, combining CIRT with temozolomide (TMZ) led to additive cytotoxicity in glioblastoma multiforme cell lines and the effect was independent of O^6^-methylguanine-DNA methyltransferase (MGMT)-expression [99]. Docetaxel has shown synergistic suppression of growth on human esophageal squamous cell carcinoma cells both in vitro and in vivo [100], and gemcitabine sensitized S-phase cancer cells, otherwise radio-resistant, to C-ions [101]. Given the increased cell killing observed in tumors, the fear of increased normal tissue toxicity when combining CIRT and chemotherapy needs to be considered and studied. The limited clinical and pre-clinical experience available encourages pursuing studies combining CIRT and dose-escalated systemic agents but extreme care should be undertaken in such studies. It remains to be determined whether the high LET C-ions may obviate the need for radio-sensitizing agents.

## 5. Clinical Experiences with CIRT

### 5.1. Brief History

Amongst the charged particles, protons and C-ions are the particles that have most widely been studied in the clinical setting. Lawrence Berkeley National Laboratory (LBNL) treated the first patients with protons, helium, and neon ions in 1954, 1957 and 1975, respectively. Unfortunately, the LBNL cyclotron was closed in 1992 after pioneering the field of charged particle therapy. Loma Linda (Fermilab) built the first proton beam radiotherapy facility with a rotating gantry in 1990. In 1994, the National Institute of Radiological Sciences (NIRS) started treating patients with C-ion beams in the Heavy Ion Medical Accelerator (HIMAC) in Chiba and thus pioneered CIRT world-wide. The Gesellschaft für Schwerionenforschung (GSI) in Darmstadt, Germany treated their first patient in 1997 with a dedicated medical irradiation room in which they developed and installed the first C-ion scanning technique as well as an inline PET camera. Germany later built the Heidelberg Ion Therapy Center (HIT) in 2009 which became the first C-ion facility with a 360° rotating gantry. The National Institute of Radiological Sciences (NIRS) installed their superconducting rotating gantry in 2015 [102]. Technological advancements in C-ion facilities, such as improved motion monitoring and raster scanning, continue to improve treatment accuracy. Currently, there are 10 charged particle radiotherapy centers actively treating patients and more in advanced planning or under development around the world.

### 5.2. Clinical Rationale

As discussed earlier, C-ions have better dose distribution properties, high RBE, and the potential for dose escalation to tumor, while still respecting normal tissue constraints [103]. Notably, ionization density, linear energy transfer, and RBE are significantly higher, lateral scatter is significantly lower, and the Bragg peak is tighter at beam edge for C-ions compared to protons (Figure 1B). Thus, C-ions have potential to be an ideal heavy particle candidate for cancer treatment, and have significant potential to overcome resistance afforded by DNA repair mechanisms. In addition, many CIRT paradigms involve hypofractionation which improves efficiency and cost-effectiveness and reduces overall treatment times [104,105]. That being said, the cost of building and maintaining CIRT centers is higher than for PhXRT or proton centers. The justification for further expanding such expensive treatments will in part be based on positive clinical data from currently active centers in Japan, Germany, Italy, and China, which would support the theoretical physical, biophysical, and biologic advantage touted in preclinical studies. In the following section, we will review the historical data as well as the accumulating clinical experiences reported using CIRT. The clinical data available thus far suggest reasonable outcomes in even hard to treat tumors, such as those that are deep seated, critically located, traditionally thought to be radio-resistant, or are recurrent and highly aggressive [106,107]. Representative current clinical trials of CIRT-based treatments are summarized in Table 1.

### 5.3. Clinical Experience by Disease Site

#### 5.3.1. Osteosarcomas and Soft Tissue Sarcomas (STS)

Most of the published data on the use of CIRT for osteosarcomas and STS patients arise from unresectable or recurrent patients who, otherwise, are considered incurable and have poor outcomes. The Bone and Soft Tissue Sarcoma Working Group from the NIRS published initial results of a phase I/II dose escalation trial on unresectable bone and soft tissue sarcomas treated with CIRT. Local control and overall survival (OS) at 3 years were 73% and 46%, respectively, without grade > 3 acute reactions [108]. NIRS also reported on their experiences with CIRT for unresectable retroperitoneal sarcomas. Tumors treated included malignant fibrous histiocytoma (*n* = 6), liposarcoma (*n* = 3), malignant peripheral nerve sheath tumors (*n* = 3), Ewing/Primitive neuroectodermal tumors (PNET) (*n* = 2), and other histologies (*n* = 10). In total, 52.8 to 73.6 GyE in 16 fixed fractions were delivered over 4 weeks. CIRT yielded 50% and 69% for overall survival and local control at 5 years, respectively. No patients developed grade 3 or higher toxicities, and no gastrointestinal tract complications were reported, with a median follow up of 36 months. These results are remarkable given that most series involving surgical therapy with or without radiotherapy have reported 5-year OS and local control of 36–64% and 28–71%, respectively [109].

For unresectable osteosarcomas of the trunk, a retrospective review of 78 patients showed that CIRT achieved a 5-year local control of 62% and overall survival of 33%. The majority of surviving patients were pain-free and able to ambulate at 5 years. Grade 3 acute and late skin reactions were seen in three patients and four patients, respectively. Grade 4 skin/soft tissue reaction requiring skin grafts occurred in three patients. Two patients experienced delayed bone fractures requiring surgery [110]. For non-surgical extremity STS, experiences from NIRS on 17 patients with CIRT yielded 5-year local control and overall survival of 76% and 56%, respectively [111]. For non-sacral unresectable spinal sarcomas, the NIRS researchers reported their experience of 47 patients treated with CIRT with impressive local control, overall survival and excellent functional outcomes as 78% of surviving patients remained ambulatory without fatal toxicities. One patient had grade 3 late skin reaction and grade 4 late skin reaction, and one patient had a grade 3 late spinal cord reaction [112]. While it is difficult to compare toxicities and outcomes between CIRT and other radiation modalities without prospective randomized studies, it is worth mentioning that in a phase II study using photon/proton irradiation for 50 patients with resectable and unresectable spine sarcomas, the grade 3 complication rate was 28% and included neuropathies, erectile dysfunction, rectal bleeding and sacral insufficiency fractures [113]. For unresectable sacral chordomas, the NIRS experience of 188 patients with CIRT achieved 77% and 81% 5-year local control and overall survival, respectively, while maintaining 97% ambulation in surviving patients. Grade 3 peripheral nerve toxicity was seen in six patients, and grade 4 skin toxicity in two patients [114]. For unresectable non-skull base chondrosarcomas, the NIRS experience of 75 patients with CIRT demonstrated 55% and 57% 5-year local control and overall survival, with four patients reported as having experienced grade 3 or 4 skin/soft tissue late adverse effects [115]. A small retrospective study comparing surgery vs. CIRT for pelvic chondrosarcomas showed similar survival but improved functional outcomes with CIRT [116]. At least one case report has shown the successful treatment of primary cardiac angiosarcoma in a young female [117]. Similarly, CIRT yielded favorable outcomes for unresectable Ewing’s sarcoma [118] and peripheral nerve sheath tumors [119] in small case series. CIRT is also being investigated currently for unresectable osteosarcomas in a prospective clinical trial [120]. Given the poor outcomes seen in these pathologies, particularly without surgery, the above reported outcomes and toxicity profiles with CIRT demonstrate significant promise.

#### 5.3.2. Head and Neck (Including Skull Base) Cancers

CIRT may provide significant benefit in head and neck cancers, given the abundance of histologies resistant to conventional PhXRT in a small anatomic estate where normal tissue sparing becomes of utmost importance. Initial data from the NIRS Working Group for Head Neck Cancers demonstrated promising outcomes with reduced acute and late reactions for CIRT in the treatment of 236 patients with head and neck melanoma, adenoid cystic carcinoma, adenocarcinoma, squamous cell carcinoma and sarcoma [121]. CIRT has shown good local control in mucosal melanomas but long-term survival is still poor, likely because of the high distant metastasis rate [122,123]. The NIRS Working Group for Ophthalmologic Tumors published the long term outcomes of CIRT for choroidal melanoma with excellent local control and eye retention rates [124]. The prospective phase II COSMIC trial which evaluated combination IMRT and CIRT for incompletely resected and inoperable adenoid cystic and other malignant salivary gland tumors showed 82% local control and 78% overall survival at 3 years with acceptable toxicity [125]. Likewise, CIRT results have been published for locally advanced squamous cell carcinoma of the ear [126], lacrimal gland carcinoma [127], mucosal malignant melanoma [128], sinonasal adenocarcinoma [129] and base of tongue adenoid cystic carcinoma [130].

Results of early phase I/II trials on skull base tumors treated with raster scanning CIRT to a median of 60 GyE from the GSI center were initially published in 2002 [131,132,133] and later updated [134,135,136,137,138]. At 5 years, local control and overall survival were 89% and 98% for chondrosarcomas and 70% and 88% for chordomas, respectively. At 10 years, local control and overall survival were 88% and 79% for chondrosarcomas and 54% and 75% for chordomas, respectively. Acute and late toxicities were mild with no grade > 3 reactions. In contrast, proton radiotherapy yielded overall survival of 62% and 91% for skull base chordomas and chondrosarcomas, respectively, with a 5-year freedom from high grade toxicity of 94% [139]. The Japanese Working Group for Head and Neck Cancers reported excellent local control, overall survival and acceptable toxicity with CIRT in the treatment of unresectable bone and STS of the head and neck [140]. Given these favorable results, many clinical trials are underway for skull base chondrosarcomas [141], skull base chordomas [142], and recurrent nasopharyngeal carcinomas [143], all tumors with less impressive outcomes with standard PhXRT.

#### 5.3.3. Prostate Cancers

The earliest dose-escalation clinical trial was reported by the Working Group for genitourinary (GU) Tumors at the NIRS with 96 T1b–T3 prostate cancer patients [144]. This study established optimal technique (shrinking fields), dose and fractionation (66 GyE in 20 fractions over 5 weeks) regimens with excellent outcomes. A follow up phase II efficacy study was then completed using the same treatment regimen for 175 patients. Notably, hormonal therapy was used in high-risk patients. Results were very promising including excellent biochemical freedom from progression, no grade ≥ 3 toxicities [145,146,147] and excellent outcomes after salvage therapy for those who fail [148,149]. Recently, health-related quality of life (HR-QoL) change patterns have been reported and revealed initial decrement in quality of life (QoL) scores with subsequent improvement to baseline levels [150]. Interestingly, shorter course regimens (57.6 GyE in 16 fractions or 51.6 GyE in 12 fractions) have also shown promising results but long-term outcomes are still pending [151,152]. The best evidence supporting the use of CIRT in prostate cancer comes from a large multi-institutional retrospective analysis of 2157 patients treated in the CIRT centers in Chiba, Gunma and Saga in Japan [153]. Over half of the patients had high-risk prostate cancer and one third had intermediate-risk cancer. All patients received hypofractionated therapy. The results were excellent for all groups, but most interestingly were the remarkable 92% and 99% 5-year biochemical recurrence-free survival and cancer-specific survival of the high-risk group. Additionally, there were no grade ≥ 3 toxicities. Clearly, CIRT brings a tremendous benefit to high-risk patients whose overall outcomes are inferior with PhXRT. Phase III clinical trials are needed but the ethical question of randomizing high-risk patient to PhXRT in such trials needs to be addressed first. Further improvements in CIRT include a further reduction in the number of fractions, spacer gel use and possibly urethral-sparing and nerve-sparing treatment planning.

#### 5.3.4. Cervical Cancers

Chemotherapy and radiotherapy (external beam plus brachytherapy) is the standard of care for locally advanced cervical cancer (LACC) [154]. Initial studies on LACC from the NIRS used CIRT (to the pelvis with boost to the gross tumor) alone without brachytherapy or concurrent chemotherapy. C-ions were initially believed to compensate for the absence of chemotherapy and brachytherapy given their high RBE, sharp Bragg peak and shorter overall treatment times, especially in the absence of data on the safety of CIRT in combination with chemotherapy or brachytherapy. The first dose escalation results were reported on 44 stage IIIB and IVA squamous cell carcinoma patients [155]. Treatment was delivered in 24 fractions over 6 weeks. Local control results were promising but there was an 18% risk of major late gastrointestinal toxicity (mostly bleeding). After further study of dosimetry as it related to toxicities, it was concluded that shrinking field technique, and limitation of bowel D_max_ to < 60 GyE should be considered. A repeat dose-escalation study established 72 GyE in 20 fractions as standard dose for LACC with promising oncologic outcomes and no grade ≥ 2 toxicities [156]. Similar results were achieved for locally advanced adenocarcinoma of the cervix [157]. The risk of para-aortic lymph node failure with or without distant metastases was high in these initial studies [158]. A significant improvement in local control and overall survival was achieved with the addition of prophylactic para-aortic field irradiation (39 GyE in 13 fractions to the extended field and 72 GyE in 20 fractions to the gross tumor) in patients with stage IIB, IIIB and IVA patients without any grade ≥ 3 toxicities [159]. Still, 30% of these patients developed distant metastases. In a separate report on 29 patients with LACC and bladder invasion, CIRT showed favorable outcomes compared to historical controls treated with PhXRT. In this study, eight patients developed grade 3 bladder complications and four patients had grade 4 rectal toxicities. Notably, four patients received concurrent weekly cisplatin without any particular increased complication rate [92].

#### 5.3.5. Hepatocellular Carcinomas (HCC)

HCC is the third leading cause of cancer death world-wide especially in areas of high hepatitis B and C prevalence. There are many treatment options for HCC but, short of liver transplantation, the benefit of all these treatments is transient and the overall survival is grave. The major limitation, in addition to the advanced nature of disease at presentation especially in developing nations, is the degree of liver cirrhosis, making any compromise of liver function (for example, radiation-induced hepatic insufficiency) in the non-cancerous liver tissue possibly fatal. Stereotactic body radiation therapy (SBRT) has shown improved tolerability and outcomes compared to conventional PhXRT [160]. Still, long-term outcomes are poor. The initial dose-escalation clinical trial from the Liver Cancer Working Group at the NIRS showed that CIRT (15 fractions in 5 weeks) is feasible, well tolerated and provides favorable outcomes for a group of stage II, III, and IV HCC patients who are recurrent after initial treatment or deemed not amenable to any other treatment approach [161]. Follow up clinical trials investigated 12, 8, 4 and 2 fractions with excellent outcomes [162,163]. The HIT German group is conducting the PROMETHEUS-01 phase I dose escalation clinical trial using raster scanning CIRT for HCC and the initial results reported appear to be promising [164,165].

Two additional studies are worth reporting. A dosimetric study from the NIRS showed improved conformity and reduced liver and bowel doses in CIRT planning compared to SBRT plans [166]. A meta-analysis by the Shanghai Heavy Ion Center demonstrated that charged particle radiotherapy had superior survival, local control and toxicity compared to conventional PhXRT. However, oncologic outcomes were comparable to SBRT [167].

#### 5.3.6. Pancreatic Cancers

Pancreatic cancer is the fifth leading cause of death world-wide. Surgery is the only curative treatment but it only applies to a small number of cases as the majority of patients usually present with advanced unresectable disease. Given their high levels of hypoxia and radioresistance, local failure remains a major form of recurrence [168] and thus, CIRT with its improved RBE and advantageous OER, is an attractive treatment approach for these patients. In a recent report from the NIRS Working Group for Pancreas Cancer, CIRT in combination with Gemcitabine was used to treat unresectable locally advanced pancreatic cancers (LAPC). Both CIRT and gemcitabine (weekly for 3 weeks) were dose escalated. Radiation was safely delivered to 55.2 GyE in 12 fractions over 3 weeks before any dose-limiting toxicity. Gemcitabine was also escalated to 1000 mg/m^2^. The 2-year survival was 48% with excellent tolerability at this dose level [91]. Gastric ulceration was uncommon after CIRT for these patients [169]. The NIRS group also tested CIRT as a pre-operative strategy for potentially resectable pancreatic cancer in 8 fractions over 2 weeks. Treatments were well tolerated and overall survival was 52% at 5 years. While distant failure was high, none of the patients experienced local failure [170]. The HIT German group is currently testing CIRT using raster scanning for advanced pancreatic cancer patients with concurrent and adjuvant gemcitabine: the PHOENIX-01 study [171].

#### 5.3.7. Glioblastoma (GBM)

GBM remains a difficult tumor to treat with poor prognosis despite using multimodality therapy which could include surgery, PhXRT and temozolomide [172]. The initial results of treatment of these high-grade gliomas were reported in 2007 by the NIRS Organizing Committee of the CNS tumor Working Group [173]. CIRT was used as a boost after conventional PhXRT to 50 Gy in 25 fractions. Concurrent nimustine hydrochloride (ACNU) was used. CIRT dose was escalated from 16.8 to 24.8 GyE in 10% incremental steps, without any acute or late grade > 2 complications. Median survival for GBM was 26 months in the high-dose arm. Based on promising data with CIRT [174], the HIT German group is comparing CIRT boost to proton boost after surgical resection and conventional chemoradiation (50 Gy with temozolomide) in patients with GBM: the phase II CLEOPATRA study [175].

#### 5.3.8. Pediatric Cancers

There have not yet been any published clinical trials specifically dedicated for CIRT in pediatric patients. Of 394 patients treated at GSI between 1997 and 2007, only 17 were ≤ 21 year old and all of them were treated for chordomas or low-grade chondrosarcomas of the skull base with excellent outcomes and minimal toxicity [176]. The HIT also published on the treatment of 36 pediatric patients with a median age of 12 years with variety of tumor types (including pilocystic astrocytoma, rhabdomyosarcoma, chordoma, osteosarcoma, angiofibroma of the nasopharynx, and adenoid cystic carcinoma) and reported favorable outcomes and acceptable toxicities [177].

#### 5.3.9. Recurrent and Previously Irradiated Cancers

Treatment of locally recurrent tumors with or without prior PhXRT is challenging given the increased tumor resistance after recurrence and the difficulty in achieving normal tissue dose constraints in previously irradiated tumors. CIRT thus is an attractive tool for such cases. The HIT team reported results of re-irradiation using CIRT for 52 patients with adenoid cystic carcinoma of the head and neck. Treatments were well tolerated without grade > 2 toxicity and with excellent control and survival rates [178]. The NIRS group reported an interesting phase I/II CIRT dose escalation study on recurrent, previously resected but not irradiated, rectal cancers. Dose was escalated to 73.6 GyE in 16 fractions over 4 weeks without grade > 3 toxicities and with excellent local control [179]. Based on initial results of re-irradiating recurrent rectal cancers [180], the HIT German group is currently investigating using CIRT as a re-irradiation strategy for previously irradiated rectal cancer: the PANDORA-01 study [181]. CIRT has been also investigated for recurrent sacrococcygeal chordomas [182], recurrent lacrimal carcinomas [127], recurrent skull base tumors [183], recurrent lung metastasis [184], and recurrent nasopharyngeal carcinomas [143], among many others [185,186]. The HIT German group is also investigating the use of intensity modulated raster-scanning CIRT re-irradiation in patients with recurrent high-grade gliomas compared to photon-fractionated stereotactic radiotherapy: the CINDERELLA trial [187].

### 5.4. Cost-Effectiveness of Carbon Ion Radiotherapy

Despite the described benefits and promising preclinical and clinical data to date, CIRT remains limited to 10 centers worldwide. The biggest obstacle for the wide-spread adoption of CIRT is the initial investment in building C-ion centers and the costs of maintenance and treatments. Only a few studies have evaluated the cost-effectiveness of CIRT. In one study, CIRT was shown to have lower overall costs compared to PhXRT for patients with skull base chordomas assuming that C-ions achieve a local control of about 70%. The incremental cost-effectiveness ratio for such treatments was estimated at about €7500 per additional life-year [188]. Similarly, CIRT was found to be a cost-effective treatment as compared to multi-modality treatment in locally recurrent rectal cancer [189]. In another analysis however, it was recommended that CIRT not be adopted as standard therapy for stage I non-small cell lung cancer given the unclear benefit, and considerable uncertainty and multiple variables in the decision-making for these patients, which leads to a questionable cost–benefit advantage [190]. However, more recent data from the NIRS showed remarkable local control for single-fraction CIRT in the treatment of stage T1 and T2 non-small cell lung cancer, which may in the future lead us to re-assess our thinking on this issue [191]. To put things into perspective, the cost of establishing a CIRT center in the United States is dwarfed by the annual revenues estimated for novel immunotherapies, some of which exceed hundreds of millions of dollars in annual sales for a single agent. In summary, continued studies on the cost-effectiveness of CIRT are needed but indeed the utility of these studies is limited by the quality of data available for each specific cancer sub-site and the high number of unknown variables that will have to be estimated in the decision-making for the management of these patients.

### 5.5. Carcinogenesis after Carbon Ion Radiotherapy

The risk of secondary malignancies after CIRT has not been reported yet likely due to the long latency of inducing second cancers and the relatively short follow up accumulated so far in treating patients with CIRT. Animal models have been sparingly used to understand the biology of heavy ion radiation-induced second cancers. In investigating the risk of secondary malignancies after CIRT, one group studied the inter- and intra-change cytogenetic damage in blood samples of patients receiving IMRT alone or IMRT and CIRT boost for prostate cancer at the HIT and GSI in 2006. Interestingly, there were no differences in the quality or quantity of chromosomal damage between the two arms. The group concluded that there is no difference in the risk of late normal tissue complications or secondary malignancies with CIRT [192]. Another group from the NIRS analyzed the genomes of T-cell lymphomas arising in mice after CIRT and PhXRT. It was noted that while these second tumors share many common mutations, large interstitial chromosomal mutations were far more common in C-ion induced secondary cancers [193]. In the absence of solid clinical data, another approach to estimate the risk of secondary cancers after CIRT relies on mathematical modeling [194]. Using the linear quadratic or the linear-no-threshold models for example, the risk of secondary breast cancers was estimated to be comparable in Hodgkin’s lymphoma patients treated with proton or carbon ion radiotherapy [195]. In any case, understanding the biology of secondary malignancies after CIRT and estimating the clinical risk of this normal tissue complication from multi-institutional patient data is a cornerstone in establishing the case for CIRT.

### 5.6. Opportunities for Further Improving Efficacy of CIRT in the Clinic

As discussed above, the contribution of different pathways (NHEJ and HR) in the repair of charged particle radiation damage is still an open question that warrants extensive mechanistic elucidation. Inducing combined lethality by selectively inhibiting the NHEJ or HR pathway components following charged particle radiation damage represents an enticing and promising combined modality therapy to enhance the treatment of primary tumors [47,48]. In addition, the determination of tumoral genetic heterogeneity coupled with an increased understanding of individual variations in susceptibility provides unique exploitable opportunities to increase the functionality and efficacy of heavy ion therapy. Along these lines, defects in the fanconi anemia/breast cancer associated-1 (FA/BRCA1) pathway proteins have been implicated as sensitizing factors for proton irradiation [196]. In addition, the increased sensitivity of the generally radio-resistant cancer stem-like cell populations (CSCs) to C-ion irradiation is also a promising avenue [197,198,199]. It is also important to determine the contribution of alternate non-homologous end joining (A-NHEJ)/microhomology-mediated end joining (MMEJ) pathway in repair of clustered DNA in non-dividing G0/G1 phase cells [200], which may shed some light on the mechanisms used to repair clustered damage in the absence of both classical NHEJ and HR. Thus, the efficacy of combining DNA repair pathway-specific inhibitors, cytotoxic chemotherapy agents, and/or immune-modulators with C-ion therapy needs to be evaluated.

## 6. Future Promises and Concluding Remarks

Heavy ion RT, including CIRT, represents a promising and revolutionary therapeutic modality that may provide additional benefits to treat tumors that are traditionally thought to be incurable with current standard modalities. Accumulated clinical evidence demonstrates that CIRT has significant advantages over conventional photon and even proton therapy, however, the lack of available experimental, pre-clinical, and patient data resulting from limited access to carbon centers and patient facilities makes a direct comparison difficult. Conversely, the relative high cost of building and maintaining a CIRT facility precludes most hospitals and universities from establishing heavy ion centers. Furthermore, due to the high cost of treating patients with CIRT, a large amount of research is necessary to identify those patient groups that will benefit the most. Thus, well designed pre-clinical studies aimed at gaining deeper insights into the radiobiological mechanisms of CIRT are necessary.

Any endeavor to increase the understanding of CIRT needs to start with greater availability and accessibility for scientists, clinicians and patients to heavy ion radiotherapy facilities, particularly in North America, where to-date none exists. Future needed studies include thoroughly investigating beam line characterization and dose distribution dynamics, including a more thorough characterization of the SOBP, and improving the accuracy of algorithms used to calculate absorbed dose and perform treatment planning. In addition, improvements in biophysical modeling can translate into LET painting-based treatment planning. Second, a more thorough understanding of the basic radiobiology of heavy ion irradiation, with special attention to DNA repair pathways, needs to be conducted and means to apply these concepts clinically need to be elucidated. Further, prospective, well designed clinical studies need to be conducted to identify patients who would garner the most benefit from CIRT. Translational efforts should include investigation of systemic therapy agents to enhance CIRT-based treatments, and determination of biomarkers of response to CIRT-based therapy. Clinically, more hypofractionation-based protocols are needed to further improve outcomes and make the case for CIRT as a cost-effective modality. In summary, CIRT is a promising and exciting modality for cancer therapeutics, with potential to overcome inherent problems related to treatment resistance including effective DNA repair mechanisms invoked in some of the most difficult to treat tumors. Furthermore, physical/biological properties of CIRT may afford not only improvement in tumor kill, but may also improve normal tissue response leading to better outcomes and tolerance, and potentially translating into an improvement in quality of life of cancer patients.

## Figures and Tables

**Table 1:** A list of selected open and/or recruiting clinical trials using carbon ion radiotherapy (CIRT) alone or in combination with other treatment modalities. This list has been compiled using information shared by Dr. Shigeru Yamada (National Institute of Radiological Sciences, NIRS) and Dr. Morihito Takita (Ion Beam Radiation Oncology Center in Kanagawa, iROCK), and from information available online on www.clinicaltrials.gov and www.umin.ac.jp/ctr. IMRT: intensity-modulated radiotherapy; JCROS: Japan Carbon-Ion Radiation Oncology Study Group; PSA: prostate-specific antigen; GM-CSF: granulocyte macrophage colony-stimulating factor.

Central Location	Trial Name (Group)	Cancer Histology/Site	Trial Design	Trial Arms	Primary End-Point
National Institute of Radiological Sciences, Chiba, Japan	JCROS-1502 (Multi-institutional)	Pancreatic cancer, T4M0	Phase II	Single arm: carbon ion therapy (55.2 GyE/12 fractions) and gemcitabine	2-year overall survival
	JCROS-1509 (Multi-institutional)	High-risk prostate cancer	Phase II	Single arm: carbon ion therapy (51.6 GyE/12 fractions) and hormone therapy	5-year biochemical relapse-free survival
		Locally advanced cervical adenocarcinoma	Phase I/II	Single arm: carbon ion therapy (20 fractions) and concurrent cisplatin	Acute toxicity and response rate
		Esophageal squamous cell carcinoma, stage II/III	Phase I/II	Single arm: preoperative carbon ion therapy (8 fractions) and concurrent cisplatin and 5-FU, followed by surgery	Acute toxicity and response rate
Gunma University Heavy Ion Medical Center, Gunma, Japan	JCROS-1505 (Multi-institutional)	Hepatocellular carcinoma, inoperable	Phase II	Single arm: carbon ion therapy (60 GyE/4 fractions or 60 GyE/12 fractions if near digestive tract)	3-year overall survival
	GUNMA-1102	Primary malignant bone and soft tissue tumor in the childhood	Phase I	Single arm: carbon ion therapy	Acute complication rate
	GUNMA-0801	Rectal cancer, post-operative pelvic recurrence	Phase I/II	Single arm: carbon ion therapy in 16 fractions	3-year local control
	GUNMA-0904	Primary malignant bone and soft tissue tumor	Phase I/II	Single arm: carbon ion therapy in 16 fractions	2-year local control
	GUNMA-0703	Hepatocellular carcinoma		Single arm: carbon ion therapy in 4 fractions	3-year local control
Heavy Ion Medical Accelerator (HIMAT), Saga, Japan	JCROS-1501 (Multi-institutional)	Lung cancer, inoperable, stage I	Phase II	Single arm: carbon ion therapy (60 GyE/4 fractions)	3-year overall survival
	HIMAT-1351	Rectal cancer, local recurrence after surgery	Phase II	Single arm: carbon ion therapy	3-year local control
	HIMAT-1341	Bone and soft tissue sarcoma, inoperable	Phase II	Single arm: carbon ion therapy	2-year local control
	HIMAT-1342	Chordoma, inoperable	Phase II	Single arm: carbon ion therapy	2-year local control
	HIMAT-1326	Pancreatic cancer, locally advanced	Phase II	Single arm: carbon ion therapy with concurrent chemotherapy	2-year overall survival
		Hepatocellular carcinoma (>3 cm)	Phase II	Single arm: carbon ion therapy in 4 fractions	3-year overall survival and cause-specific survival
		Hepatocellular carcinoma (≤3 cm)	Phase II	Single arm: carbon ion therapy in 2 fractions	3-year local control
		Non-small cell lung cancer, central, stage I	Phase II	Single arm: Carbon ion therapy (12 fractions)	3-year local control
		Non-small cell lung cancer, peripheral, stage I	Phase II	Single arm: carbon ion therapy (4 fractions)	3-year local control
Ion Beam Radiation Oncology Center in Kanagawa (iROCK), Kanagawa, Japan	iROCK-1601LI and iROCK-1604LI	Hepatocellular carcinoma	Phase II	Single arm: carbon ion therapy in 2 or 4 fractions	3-year local control
	iROCK-1504LU	Non-small cell lung cancer, small, peripheral, stage IA	Non-randomized, phase II	Arm 1: carbon ion therapy Arm 2: surgical resection	5-year overall survival
	iROCK-1605PA	Pancreatic cancer, locally advanced	Phase II	Single arm: carbon ion therapy (12 fractions) and gemcitabine	3-year overall survival
	iROCK-1603HN	Mucosal malignant melanoma of the head and neck	Phase II	Single arm: carbon ion therapy (16 fractions) combined with anti-tumor agents	3-year overall survival
	iROCK-1501PR	Prostate cancer, T1c-T3N0M0	Phase II	Single arm: carbon ion therapy (12 fractions)	5-year biochemical relapse-free survival
		Prostate cancer, T1b-T3N0M0	Phase II	Single arm: carbon ion therapy (12 fractions) with hormone therapy	5-year biochemical relapse-free survival
		Non-squamous cell carcinoma of head and neck (no melanoma nor sarcoma)	Phase II	Single arm: carbon ion therapy (16 fractions)	3-year local control
Heidelberg University, Germany	HIT-1	Chordoma of the skull base	Randomized, phase III	Standard arm: proton therapy Experimental arm: carbon ion therapy	Local-progression-free survival
	CSP12C	Low and intermediate grade chondrosarcoma of the skull base	Randomized, phase III	Standard arm: proton therapy Experimental arm: carbon ion therapy	Local-progression-free survival
	ISAC	Sacrococcygeal chordoma	Randomized	Standard arm: proton therapy Experimental arm: carbon ion therapy	Toxicity
	ACCEPT	Adenoid cystic carcinoma	Phase I/II	Single arm: cetuximab and IMRT plus carbon ion boost	Toxicity
Shanghai Heavy Ion Center		Hepatocellular carcinoma	Non-randomized, phase II	Single arm: Carbon ion therapy with GM-CSF	Progression-free survival
		Hepatocellular carcinoma	Phase I	Carbon ion therapy or carbon plus proton therapy depending on proximity to bowel	Toxicity
		Localized prostate cancer	Phase I/II	Single arm: carbon ion therapy	Toxicity
		Oligo-metastatic prostate cancer	Phase II	Single arm: carbon ion therapy to prostate plus chemotherapy or hormonal therapy	Time to PSA relapse
		Locally recurrent nasopharyngeal carcinoma	Non-randomized, phase I/II	Single arm: carbon ion therapy, 2.5 GyE or 3 GyE per fraction	Toxicity
National Center of Oncological Hadrontherapy (CNAO), Italy		High risk prostate cancer	Non-randomized, phase II	Single arm: carbon ion boost followed by conventional photon RT	Toxicity
	SACRO	Localized sacral chordoma	Randomized	Standard arm: surgery Experimental arm: radiotherapy including option for carbon ion therapy	Relapse-free survival
